# Experiences of implementation of the group-based adaptation training intervention for patients with chronic somatic illnesses or disabilities among multi-professional teams in specialized healthcare

**DOI:** 10.1080/07853890.2023.2253725

**Published:** 2023-09-11

**Authors:** Heidi Siira, Maria Kääriäinen, Ulla Jämsä

**Affiliations:** a Research Unit of Health Sciences and Technology, Faculty of Medicine, University of Oulu, Oulu, Finland; b Department of Medical Rehabilitation, Wellbeing Services County of North Ostrobothnia, Oulu University Hospital, Oulu, Finland

**Keywords:** Adaptation training, multi-professional, rehabilitation, specialised healthcare, chronic illness, disability

## Abstract

**Objective:**

To describe the experiences of multi-professional teams of implementation of group-based adaptation training intervention for patients with chronic somatic illnesses or disabilities in specialized healthcare.

**Materials and methods:**

Multi-professional teams (*n* = 7) implementing adaptation training courses for chronically ill patients in specialised healthcare were interviewed between 09/2020 and 12/2021. The themes for thematic group interviews were based on the standard protocol implementation of adaptation training in specialised healthcare, including planning, implementation and evaluation of the adaptation training courses. The interviews were audio-recorded and transcribed. The data were analysed using inductive content analysis.

**Results:**

The experiences of multi-professional teams involved using pedagogical methods, providing guidance and counselling to support the rehabilitation process, ensuring opportunities for peer support, and supporting the course participants’ involvement and activities in everyday life.

**Conclusions:**

Healthcare professionals should use pedagogical methods in reflective guidance and counselling to promote client-oriented approach in supporting adaptation. Their competence in pedagogy needs to be build and maintained by continuous education. Multi-professional teams need to ensure sufficient and versatile conditions for peer support and involvement of family members by creating open and trusting atmosphere, unhurried encounters, discussions, different and varying ways of working. Adaptation training can strengthen the self-efficacy of participants and help them shift their attention from illness and disability to thoughts of the future. Adaptation training can support active and meaningful daily life in a changed life situation.

## KEY MESSAGES

Pedagogical methods are useful in multi-professional group rehabilitation interventions.

Multi-professionalism is essential to providing meaningful guidance and counselling to support the rehabilitation process of patients with long-term illnesses and disabilities.

Application of provided knowledge and counselling to everyday life as well as the involvement of family members and taking advantage of the possibilities of peer support, enables active agency in daily life and rehabilitation.

## 1. Introduction

One person in three needs rehabilitation at some point in life due to illness, injury or disability [1]. Morbidity causes changes to physical, psychological, cognitive and social health. Previous literature indicates that people with somatic chronic diseases have functional disabilities that affect their participation in social life [2–4], daily life activities [5,6] and mental functions [7]. In addition, adults with chronic conditions entail a high burden and cost for the healthcare system [8].

The periods of care and treatment in healthcare have become shorter, which highlights the importance of patient counselling. Counselling is key in helping a patient manage and cope with a new situation, such as receiving a diagnosis or becoming injured or disabled. According to previous literature, patients want more information about diseases, rehabilitation possibilities and peer support and they value a holistic relationship with healthcare professionals [9].

Group rehabilitation interventions can improve patients’ self-management and abilities to cope with their illnesses. According to previous studies, group rehabilitation interventions provide important skills related to various areas of life [10,11]. Peer support offered by groups enables sharing experiences, exchanging ideas and finding solutions to daily-life problems [12,13]. The goal of group rehabilitation interventions is to find the means and courses of action that lead to patient empowerment [14].

Adaptation training intervention, which is part of rehabilitation counselling and medical rehabilitation, is a Finnish version of psychosocial group rehabilitation interventions. It is a supportive group intervention delivered by a multi-professional team including a doctor, nurse, rehabilitation counsellors, psychologist, physiotherapist, occupational therapist, social worker, and other healthcare professionals from different special medical fields. The composition of the multiprofessional group is formed based on the needs of illness or disability in question. Healthcare professionals of specialised healthcare that are involved in adaptation training interventions work closely with non-profit patient organizations to make guidance and counselling wide ranging. Standard protocol implementation of these interventions are residential courses, which usually last for three days. The intervention’s objective is to provide the participants with knowledge, psychosocial support, and peer support during the course. Working methods include lectures, group work and discussions, functional groups, and separate program for family members. The long-term goal is to motivate participants to maintain and improve their functional abilities, find new operation models and abilities to live with disease and better cope with everyday life. During adaptation training, participants have the opportunity to deepen and strengthen the knowledge and skills developed during previous counselling [15].

Multidisciplinary teams need a common framework to secure the best outcomes for the adaptation training provided as a group rehabilitation intervention [16]. The International Classification of Functioning, Disability and Health (ICF) provides a uniform language framework for multi-professional work, helping different professionals understand functioning in the same way [17]. According to previous studies, the ICF framework is a useful tool that can facilitate professionals’ assessments of functioning [18,19] and multi-professional teams’ planning of objectives and interventions for rehabilitation [20]. Earlier studies have reported positive impacts group rehabilitation interventions [21,22]. However, multidisciplinary teamwork in adaptation training has been insufficiently researched nationally and internationally.

The objective of this qualitative study is to describe the experiences of multi-professional teams of implementation of normal protocol group-based adaptation training intervention for patients with chronic somatic illnesses or disabilities among in specialized healthcare. This study addresses the gap in knowledge regarding multidisciplinary healthcare teams’ experiences of delivering group adaptation training intervention from a holistic and person-centred point of view. A qualitative approach was chosen to deeply understand the experiences of the professionals. Group interview method was chosen for data collection to enable reflective discussion and sharing in multi-professional teams. Aim of the study was to produce new knowledge for development of adaptation training implementation and provision. The study findings can also be used to develop group rehabilitation practices and counselling methods to best support rehabilitees’ abilities to function and improve their quality of life and adaptation to living with illness and/or disability. The research question was as follows: What kind of experiences do multi-professional teams have of the implementation of normal protocol adaptation training interventions for chronically and somatically ill or disabled patients in specialized healthcare?

## 2. Materials and methods

### 2.1. Context

This study forms part of a larger research project ‘Adaptation training interventions in specialized healthcare promoting functional ability and quality of life’ being conducted at Oulu University Hospital in Northern Finland between 2020 and 2024. The data for this study were gathered between 09/2020 and 12/2021 through seven thematic group interviews with multi-professional teams that implemented adaptation training courses in specialised healthcare for chronically ill patients with rheumatic diseases, cancer, neurological diseases and visual and hearing impairment.

### 2.2. Data collection

The interviewed groups were recruited in the order in which the courses were implemented, from as many medical specialties as possible. The average size of the interviewed groups was four professionals. The professionals were rehabilitation counsellors, nurses, doctors, social workers and psychologists with professional backgrounds who worked in specialised healthcare. The themes of the interviews were based on the standard protocol of the implementation process, which involves the planning, implementation and evaluation of adaption training courses. The professionals’ work experiences in adaptation training ranged from a few months to decades.

The interviews took place on the hospital’s premises in available meeting rooms that were easy to reach for the participants. One interview took approximately one hour. The interviews were recorded and transcribed using Microsoft Word software. The interviews were anonymised by replacing interviewed group´s names with numbers to anonymise the data. The raw data consisted of 69 single-spaced pages of text with a 12-point font.

### 2.3. Analysis

The data were analysed using inductive content analysis [23,24]. First, the material was read several times to form a global understanding and to get a sense of the data. An expression of a theme was selected as the unit of analysis. First, original expressions corresponding to the research question were identified, listed and simplified. Second, subcategories were formed by grouping and combining expressions with the same meaning. Abstraction of the data continued by combining subcategories to form upper categories and then main categories. All categories were named according to their content.

The Northern Ostrobothnia Hospital District approved the study, and the Regional Ethics Committee provided a favourable assessment of it (reference number:3/2020). Good research ethics [25] and scientific practices [26] were followed throughout the study. The participants in the study were volunteers and gave their informed consent to take part in the study at the beginning of the interview. According to the principles of the General Data Protection Regulation, the gathered data were processed, analysed and stored confidentially so that no individual could be identified.

## 3. Results

**Figure 1.**
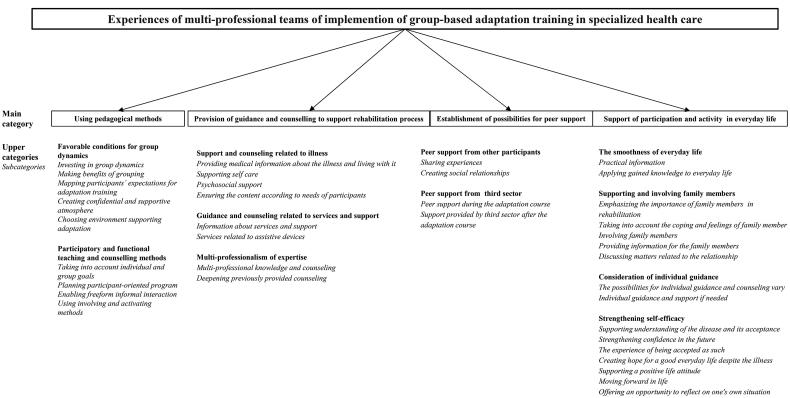
The results presented as sub, upper and main categories according to the inductive content analysis.

The following four main categories described the experiences of implementation of the adaptation training interventions: (1) Using pedagogical methods, (2) Provision of guidance and counselling to support the rehabilitation process, (3) Establishment of possibilities for peer support and (4) Support of participation and activity in everyday life. The results are presented as sub, upper and main categories according to the inductive content analysis in Figure 1.

### 3.1. Using pedagogical methods

The main category – Using pedagogical methods – involved the notions of favourable and functional conditions for group dynamics. The counselling and guiding methods were experienced participatory and rehabilitative. The focus of this category was on interaction, functionality and involvement.

To create and maintain positive group dynamics, the professionals established a confidential environment and a supportive atmosphere for group work. They fostered the feeling that all participants worked together for a common goal. The purpose of the groupwork was to instil a sense of togetherness.

We emphasise that participants can speak confidentially. They don’t need to speak if they don’t want to. I think that it is very important to work together for a common goal. (G4)

When applying to the course, the participants have set their own aims for adaptation training. In the beginning of the course the participants work together in small groups to create common goals based on individual aims for the adaptation training. All the professionals were familiar with individual aims and created group goals, which they considered in their workshops, as exemplified by the following description:

When they apply for the course, they fulfil their own goals [for the application form]. (G3)

Then they create the goals in small groups and the goals are in view on the wall. The lecturers will see what themes are hoped for and can steer the discussion the right way. (G3)

The professionals created many possibilities for the participants to get to know one another in different workshops and group sessions and in their free time. The professionals used different methods to encourage the participants to take part in the discussions. They encouraged the participants to share their opinions and experiences. The discussions were experienced informal, and the participants knew that sharing was voluntary.

The discussions in the group are steered by the participants’ needs. The professional has the skill to involve participants so that they discuss a lot and they change their experiences. (G1)

Before implementing the adaptation training course, the professionals felt important to ensure that the group would be homogenous in terms of the participants’ characteristics, and that the participants would have the capacity for rehabilitation and would benefit from the course.

The participants should be of almost the same age, representatives of different sexes and the diagnosis would have been given within the last year. In addition, participants can bring along their trusted family member, friend or child. (G3)

The professionals approached the participants as valuable human beings, and humour was considered as an instrument to create a relaxed atmosphere. The adaptation course was wanted to organize outside the hospital so that the environment would be pleasant.

We usually get positive feedback that we are relaxed and genuine but still professional. We are experts, we know our job and we laugh a lot. Still, we don’t lose our professionalism. It emphasises that we live in the moment, giving hope to the participants. (G3)

To involve participants, the professionals emphasised and said they used multiple participatory and rehabilitative counselling and guiding methods. In addition, the premises were arranged to be suitable for interaction. One premise used for the counselling methods courses was a learning café. To inspire participants to apply the knowledge to their own life situations, the professionals had participants do different exercises. Between counselling sessions, there is room for relaxation sessions.

We arrange premises suitable for interaction; for example, tables are prearranged so that participants see each other’s faces. (G6)

Functional methods like the learning café serve the group better through doing. (G3)

Participants are offered an opportunity to discuss their situation with professionals and ask about things that occupied their minds. The adaptation training and discussions were based on the participants’ needs and the applicability of the solutions to the participants’ own situations.

Adaptation training is a continuation of treatment. Participants ask questions about the couple’s relationship and future and other things that occupy their minds. They have more questions than during a hospital visit. (G5)

### 3.2. Provision of guidance and counselling to support rehabilitation

The main category of providing guidance and counselling to support rehabilitation emphasised multi-professional expertise and practicality. The programme provided extensive knowledge about illness and different services, including the use of assistive devices. The purpose was to build on previous counselling work.

The programme was versatile, and the aims were patient centred. Doctors’ lectures and workshops imparted medical knowledge to provide support for self-care and new viewpoints for living with disabilities. Professionals shared information regarding services and social security benefits. Participants received different written materials and handbooks. Professionals in multiprofessional teams worked in close cooperation with each other in implementing adaptation training courses.

It is important that we support them and tell them what kind of services they can get so that they can cope at home. (G2)

Expertise is based on multi-professional work as well as non-medical viewpoints. (G1)

The professionals stated that work progressed practically, according to the participants’ aims and questions. Knowledge was integrated to suit the participants’ daily lives. Connections were made to different everyday situations.

Participants have to apply knowledge to their own daily lives and think about how to use this information. (G2)

Psychosocial and psychological support played an important role in adaptation training. The participants had an opportunity to process the knowledge acquired during the workshops, in addition to working through their feelings.

They need a lot of time to process their feelings. It is a long process. (G4)

### 3.3. Establishment of possibilities for peer support

The main category of ensuring possibilities for peer support involved the notion of peer support from other participants or from non-profit [third sector] organizations, both during and after the course. Peer support played a significant role in adaptation training. All professionals talked about the participants’ wishes and aims during the course. According to the professionals, it was important for the participants to be able to share their own experiences and hear each other’s stories.

Really, their most important aim is to get peer support. (G3)

In adaptation training course, the participants met other people whose situations were similar. They shared their experiences and received peer support, which helped them adjust to their own illnesses. They also gave practical tips to each other. Quite often, the participants formed friendships. After the course, the participants might have been so connected that they exchanged their phone numbers and arranged meetings for further peer support.

They always hoped that there would be more free time to talk to each other. The participants receive support from the professionals and peer support from other participants. (G6)

The non-profit organizations also played an important role in providing peer support after the adaptation training.

Participants learn about third-sector organisations, and they are guided to these organisations and their services. (G3)

### 3.4. Support of participation and activity in everyday life

The main category of supporting participation and activity in everyday life addressed the issue of facileness in daily life. The guidance and knowledge were practical, which supported everyday performance. Versatile support strengthened the participants’ self-efficacy. The important task was to support and involve family members in the adaptation process. Individual counselling was provided in the lobby, during breaks between courses.

They constantly have to reflect the information received to their own everyday life, how this happens in my life and what I do with this information. They should have something concrete to have at least some kind of plan in mind. (G2)

The professionals emphasised the importance of family members in rehabilitation and striving for a common goal. The professionals stated that family members’ well-being was taken into account. Family members received information, and they had separate discussion groups in which they had opportunities to share their experiences and feelings. Matters related to relationships were also discussed.

Regarding family members, they have to remember and take into account several points in relation to their own well-being. (G3)

The participants could experience optimism, hope and understanding. Family members or friends could also understand the nature of the disability. There were lectures and workshops regarding feelings related to illness and disability. There were also exercises that addressed this aspect.

When they have hope, they begin to take care of themselves. (G1)

The professionals considered the participants’ needs for individual guidance. However, the possibilities for offering such guidance were limited.

It is possible that professionals have private discussions with some participants. (G7)

The professionals assumed that adaptation training strengthened the participants’ self-efficacy because through adaptation training intervention a better understanding of the illness, its acceptance and confidence for the future can be facilitated. The participants were encouraged to be themselves despite their chronic conditions.

They can get the feeling that they can look forward to life and receive help. It is not the end of the world to become disabled and live with a disability. (G7)

The professionals stated that the goal of adaptation training was to ‘let the illness go.’ Consequently, the participants would feel more at ease with themselves and could move forward with their daily lives.

They can process that they had the disability and now they have recovered. This process includes all kinds of feelings and changes in the state of health, but now they can be safe from that. (G5)

The professional experienced that adaptation training course makes it possible to start adaptation or to proceed with it. During the training, the participants had a chance to calm down and contemplate their lives. They were away from their home environments and everyday requirements.

The adaptation training course goes on for many days, so they have a chance to calm down and look at their own situations, which permits adaptation to new life situations. (G4)

## 4. Discussion

This study described multi-professionally implemented adaptation training in specialised healthcare. In addition to the traditional biomedical perspectives used in medical rehabilitation, the adaptation training courses also employed a holistic perception of humans and supported the participants’ abilities to function by empowering them and taking their resources into account.

This study considered the issue of professionals using pedagogical methods to implement participatory and functional teaching and counselling methods, as well as to create favourable and functional conditions for group dynamics. The professionals applied a socio-constructivist view of learning [27] when integrating theoretical knowledge to participant´s daily lives, and to motivating participants to active agency in their rehabilitation process. This could be observed in group discussions and in how professionals involved participants to reflection. In rehabilitation practices, attention should be paid to the pedagogical skills of the professionals implementing group-based rehabilitation to support patients’ adaptation to illness and/or disability. Further improving the competence of professionals in consciously using pedagogical methods would support an even more goal-oriented transfer of knowledge to everyday life.

The adaptation training provided wide-ranging and in-depth multi-professional guidance and counselling. Overall, based on the results, ICF was the main principle and theoretical structure guiding the work. Professionals had ICF knowledge which they applied and integrated to implementation of adaptation training to support the participants’ abilities to function. The focus was on supporting participation and activity in everyday life, as well as ensuring access to peer support. Environmental factors were also considered. Although the goals of the adaptation training were group based, individual aims were also considered and encouraged. The multidisciplinary of the teams implementing adaptation training is a factor that helps ensure that functional capacity is assessed and supported from a holistic perspective by considering all factors affecting such capacity (i.e. physical, psychological, environmental, social and personal).

Guaranteeing access to peer support was essential in multi-professionally implemented adaptation training. The existing literature highlights the importance of peer support in promoting participation and improving quality of life [28]. According to the findings of this study, the multi-professional teams established an environment of trust and encouraged course participants to actively take part in discussions to enhance peer support and the sharing of experiences. Through conversation and mutual sharing, it was important to create a common goal. Previous studies have highlighted the importance of goal setting, the sense of security and open interaction in building group dynamics [10] and have emphasised the importance of offering peer support respectfully while taking into account individual needs [29].

Moreover, previous studies have also shown that, in self-management interventions, peer support is essential for sharing experiences, social comparison, learning and motivation [11–13]. Rehabilitation interventions with psychosocial support facilitate physical, mental and social performance, along with the ability to function independently [30–32] and take responsibility for personal situations [9]. Peer support is beneficial to both persons with disabilities and their family members. Involving family members is an opportunity which requires more attention in future research and in implementation of such adaptation interventions since family members can influence participants perceptions of self-efficacy and reinforce behavior change.

One of the main goals of the multi-professionally implemented adaptation training was to support participants in finding ways to live meaningful lives despite illness or disability. This was done by strengthening self-efficacy and encouraging the confidence the participants need for managing personal situations, coping with everyday life and participating in society. According to Bandura’s theory [33], self-efficacy is a person’s confidence in their ability to perform a task and an individual’s belief in their own capacity to achieve things. Self-efficacy is also related to self-control and the ability to modulate one’s behaviour to reach goals. Perceived self-efficacy influences one’s management of stress and challenges and the amount of effort needed to reach one’s goals [33]. Self-efficacy is an important factor in increasing patients’ quality of life [34] and encouraging self-management [35]. With knowledge and support, one can increase a patient’s self-confidence, promote coping behaviours [11] and improve motivation [36]. As this study showed, participants found hope for the future, which facilitates better psychosocial adjustment and higher levels of self-efficacy while decreasing the levels of depression and the importance of perceived problems [37]. Earlier studies showed that in multidisciplinary rehabilitation, patients develop stronger self-efficacy and higher chances of coping [38].

In future research, the implementation of adaptation training should be studied qualitatively from the perspectives of patients and their family members. The expectations and goals set by patients and the perceived benefits of adaptation training should be investigated, in addition to examining the role and meaning of adaptation training in the continuum of the rehabilitation process. The pedagogy of adaptation training and rehabilitation counselling should also be investigated.

## 5. Trustworthiness and limitations

The trustworthiness of the study is discussed according to the criteria of credibility, transferability, dependability, confirmability and authenticity [39]. The data-driven inductive content analysis was highly suited for the collected data. Open and genuine dialogue in the research group throughout the research process increased the dependability of the analysis. The researchers were familiar with the studied area because one of them had worked as a rehabilitation counsellor in specialised healthcare and the other was working as a specialist in the field. Both researchers had pedagogical training and experience in leading groups of patients. Their preliminary understanding of adaptation training and approach to disability adaptation helped the research process but did not affect the results and conclusions in any way. The transparency, consistency and confirmability of the analytic process is described in Table 1.

**Table 1.** The transparency, consistency and confirmability of the analysis process.

Simplified expressions	Subcategory	Upper category	Main category
Grouping process of participants G1	Investing in group dynamics	Favorable conditions for group dynamics	Using pedagogical methods
Efforts are made to support grouping G3			
The goal is to work together for a common goal G4			
Grouping increases discussion and exchange of experiences G1	Making benefits of grouping		
Increasing activity through grouping G1			
Successful grouping helps the course participants to keep in touch after the course G1			
Interaction of course participants with each other in the non-supervised activities of the course G1			
Expectations are asked in the group G2	Mapping participants´ expectations for adaptation training		
Expectations are asked right at the beginning of the course G1			
Creating a confidential atmosphere to support the success of the course G4	Creating confidential and supportive atmosphere		
A confidential atmosphere enabling discussion G4			
Relaxed atmosphere supports the process G7			
Relaxed atmosphere supports the group’s activities G3			
Activity is voluntary G2			
Encouragement G2			
Relaxation G1			
Using humor G3			
The course offers a break from everyday life G4	Choosing environment supporting adaptation		
The place and environment of the course supports rehabilitation G2			
The goals of the course participants guide the content throughout the entire course G3	Taking into account individual and group goals	Participatory and functional teaching and counseling methods	
Course goals are set by participants G2			
Course participants write their own goals reflecting to the content of the course G2			
The course participants think about the goals of the course in small groups G2			
Working in accordance with the goals set by the course participants G5			
No specific goals are set for the course G1			
Targeting the conversation according to preliminary information available G4	Planning participant-oriented program		
Taking course feedback into account G1			
Progressing on the terms of the course participants G5			
The day’s program is flexible according to the needs of the course participants G4			
The working methods take into account the characteristics brought about by the participants´ illness G4			
Background information of participants is gathered to the doctor’s lecture is by a questionnaire G1			
The course program has a specific framework G4			
Enabling conversations G1	Enabling freeform informal interaction		
Course participants have the opportunity to ask the professionals and comment G5			
Free discussion G1			
Course participants are activated to ask all kinds of questions related to the disease G4			
Free-form discussions and questions G4			
Discussion along with the lectures G6			
Functional exercises to support the knowledge part of the course program G3	Using involving and activating methods		
Functional groups instead of lectures G3			
Functional tasks to activate the course participants G3			
Involvement of course participants through doing G3			
Learning cafe is a participant-oriented way of working G4			
Doing exercises G6			
The Learning-cafe activity is used to involve the course participants G2			

Recording the interviews increases the reliability of the study. However, the fact that the interview topics were not pretested may have decreased the reliability of our study. Some of the respondents were familiar with the researchers from previous multi-professional cooperation experiences on the premises of the same hospital. Even though all multi-professional teams were not able to participate in the interviews, the data were rich and saturated. The participants’ quotations provided in the article support the authenticity of the study. They were chosen systematically by the last author, who saw them as relevant to the research questions. The results are transferable to similar settings and multi-professional teams that provide adaptation training.

One limitation of the study was that three out of seven multi-professional teams provided adaptation training to neurological patients. Different professions were well represented in the interviewed teams, but not all multi-professional teams that organize adaptation training courses were not interviewed. This was due to adaptation training courses being cancelled during the COVID-19 pandemic. In addition, the size of the interviewed groups varied. The multi-professional teams reflected the reality of adaptation training provision in specialised healthcare. Practices were different according to the various medical fields providing adaptation training. For example, the working experience of professionals in adaptation training varied, as did the practice of referring the patients to the course. This variation contributed to the richness of the material and increased the trustworthiness of the results.

## 6. Conclusion

The results of this study strengthen the understanding that adaptation training courses should be carried out by multi-professional teams with comprehensive guidance and support to promote rehabilitees adaptation process. Both the goals of the participants and the entire group should be taken into account when providing counselling and dealing with issue related to adaptation. Adaptation training interventions require enough time as well as favourable and versatile conditions. Open and trusting atmosphere, unhurried encounters, discussions, different and varying ways of working enable peer support and involvement of family members. Healthcare professionals in multi-professional teams emphasise pedagogical methods to provide information, support, and counselling to participants, but they also need continuous education to maintain their competence in pedagogy. Adaptation training can strengthen the self-efficacy of participants and helps them shift their attention from illness and disability to thoughts of the future and to rewarding, independent, active, and meaningful lives with and despite the disease and disability.

## Data Availability

The data that support the findings of this study are available from the corresponding author, [HS], upon reasonable request.
